# Pseudodiabetes—not a contraindication for metabolic interventions

**DOI:** 10.1038/s41419-019-2007-1

**Published:** 2019-10-10

**Authors:** José Manuel Bravo-San Pedro, Valentina Sica, Guido Kroemer

**Affiliations:** 1 INSERM U1138, Centre de Recherche des Cordeliers, Sorbonne Université, Université de Paris, Paris, France; 2 Team “Metabolism, Cancer & Immunity” labellisée par la Ligue contre le Cancer, Paris, France; 3 Cell Biology & Metabolomics platforms, Gustave Roussy Cancer Campus, Villejuif, France; 4 Pôle de Biologie, Hôpital Européen Georges Pompidou, AP-HP, Paris, France; 5 Suzhou Institute for Systems Medicine, Chinese Academy of Sciences, Suzhou, China; 6 Karolinska Institute, Department of Women’s and Children’s Health, Karolinska University Hospital, Stockholm, Sweden

**Keywords:** Metabolic disorders, Endocrine system and metabolic diseases

## Abstract

Type-2 diabetes is characterized by glycosuria, hyperglycemia, glucose intolerance, hyperinsulinemia, and insulin resistance. One or several among these alterations are also found after starvation, ketogenic diet, and pharmacological treatment with rapamycin or antibody-mediated neutralization of the obesogenic factor ACBP/DBI. Thus, a variety of metabolic interventions that improve metabolic health can induce a transient state of “pseudo-diabetes”.

Type 2 diabetes, which is typically associated with obesity and advanced age, is an initially indolent disease accompanied by changes in laboratory parameters, in particular, glycosuria (presence of glucose in urine), hyperglycemia (increased plasma glucose concentration), glycated hemoglobin (A1C test), glucose intolerance (measured by the glucose tolerance test that determines the capacity to control glycaemia upon oral glucose challenge), hyperinsulinemia (increased plasma insulin concentration), and insulin resistance (measured by means of the insulin tolerance test that quantifies the reduction in blood-glucose levels after insulin injection). Since diabetes is one of the most important factors causing a reduction of health span and lifespan in the global population, drugs that may cause diabetes as a side effect (http://www.diabetesincontrol.com/drugs-that-can-affect-blood-glucose-levels/) are usually submitted to special scrutiny when they are administered to patients.

The conservative treatment of type 2 diabetes consists in weight loss programs, often based on fasting programs or ketogenic diet (which is a carbohydrate-poor, high-fat, and sufficient-protein diet) combined with physical exercise. Of note, caloric restriction and ketogenic diet also extend health span and lifespan in all animal species investigated in this respect, supporting beneficial effects on general metabolism beyond the prevention or treatment of type 2 diabetes^1,2^. While caloric restriction extends lifespan through the induction of autophagy, the most important cytoplasmic rejuvenation pathway^3,4^, it is not yet known whether ketogenic diet requires autophagy induction to be efficient. However, it is well established that the antidiabetic effects of endurance exercise are mediated by autophagy induction^5^. Moreover, pharmacological induction of autophagy in mice by spermidine, an inhibitor of the acetyltransferase EP300, reduces the propensity of the animals to put on weight and to become diabetic when they are placed on a high-fat diet. This anti-obesity and antidiabetic effect of spermidine is lost in mice that bear a partial autophagy defect due to the homozygous knockout of *Atg4b*^6^, and similarly the capacity of spermidine to avoid organismal or cardiovascular aging fully depends on autophagy^7^. Of note, another pharmacological autophagy inducer, rapamycin, an inhibitor of mechanistic target of rapamycin complex 1, prevents insulin resistance caused by nutrient infusion in humans and diminishes signs of type 2 diabetes in mice^8^. Rapamycin is known to mediate its health-promoting effects via the induction of autophagy^1^. Finally, neutralization of the protein acyl-CoA binding protein (ACBP, also known as diazepam-binding inhibitor, DBI) by antibodies induces autophagy and reduces the propensity of mice to develop glucose intolerance under high-fat diet^9,10^. Thus, as a general pattern, it appears that stimulation of autophagy has antidiabetic and general antiaging effects. The common denominators of many of the aforementioned antidiabetic treatments are an increase in ketone bodies (acetoacetate and 3-hydroxybutyrate) alone or combined with an increase in autophagy. Ketosis (an increase in circulating ketone bodies) is observed after starvation^4^, in the context of ketogenic diets^2^, but also after deletion of the gene coding for ACBP/DBI^9^. Starvation, exercise, spermidine, and rapamycin all potently induce autophagy. However, the links between ketogenic metabolism and autophagy have not been established, requiring further in-depth investigation of these phenomena.

In spite of the undoubtable antidiabetic effects of the aforementioned interventions, many of them induce a phenomenon that can be referred to as “pseudo-diabetes” (Fig. 1), namely a change in laboratory parameters that are indicative of diabetes: glycosuria, hyperglycemia, glucose intolerance, hyperinsulinemia, and insulin resistance, as recently brought up by Blagosklonny^8,11^. Indeed, the French physiologist Claude Bernard was the first to note in 1846 that rabbits that were on a starvation diet developed glycosuria after having been refed with carrots, hence developing a “starvation diabetes”. Similarly, ketogenic diets induce glucose intolerance and insulin resistance in mice, a phenomenon that is reversed upon cessation of the diet. Hence, ketogenic diets also induce pseudo-diabetes^11^. In response to chronic rapamycin treatment, a mild hyperglycemia, glucose intolerance, and insulin resistance is observed, again revealing signs of pseudo-diabetes^8^. Finally, injection of monoclonal antibodies that neutralize ACBP/DBI causes a mild hyperglycemia that mediates the anorexigenic (appetite-suppressing) effects of this maneuver. This hyperglycemia results from enhanced lipolysis, generating glycerol from triglycerides and subsequent use of glycerol for gluconeogenesis^9^. Thus, ACBP/DBI neutralization again induces some features of pseudo-diabetes. At this point, it is not known, however, whether these features of pseudo-diabetes are secondary to ketosis and autophagy induction or whether they can occur independently.

**Fig. 1:**
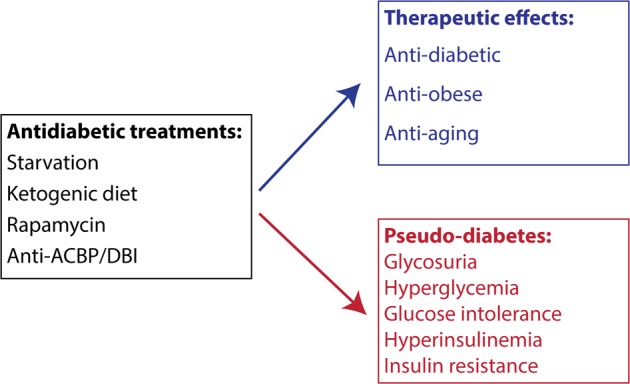
Pseudo-diabetes and its implications

The aforementioned observations generate an intriguing paradox. Several established treatments of type-2 diabetes (exemplified by fasting and ketogenic diet) and several experimental therapies (exemplified by rapamycin and ACBP/DBI neutralization) cause signs of pseudo-diabetes. Hence, the same therapeutic procedure can induce (generally transient) changes in laboratory parameters that suggest the induction of a pre-diabetic or diabetic state, yet have long-term preventive and curative effects on type-2-diabetes. Although it may premature to postulate that the induction of pseudo-diabetes is the cause of the antidiabetic effects of these therapies, it appears clear that special attention has to paid to the overinterpretation of pseudo-diabetic features as a no-go in the future development of antidiabetic, anti-obesity and antiaging drugs. If pseudo-diabetes was a contraindication for antidiabetics, regulatory instances such as the Food & Drug Administration would not have allowed drastic caloric restriction or ketogenic diets to be developed for the treatment of type-2-diabetes. Applying a similar logic, drug-based therapies should not be blocked in their development as potential antidiabetics because they cause signs of pseudo-diabetes.
